# Inhibition of Hippocampal Neurogenesis Starting in Adolescence Increases Anxiodepressive Behaviors Amid Stress

**DOI:** 10.3389/fnbeh.2022.940125

**Published:** 2022-07-05

**Authors:** Rachelle Larivee, Natalie Johnson, Natalie R. Freedgood, Heather A. Cameron, Timothy J. Schoenfeld

**Affiliations:** ^1^Department of Psychological Science and Neuroscience, Belmont University, Nashville, TN, United States; ^2^Section on Neuroplasticity, National Institute of Mental Health, National Institutes of Health, Bethesda, MD, United States

**Keywords:** adolescence, adult neurogenesis, anxiety, depression, stress

## Abstract

Stressors during the adolescent period can affect development of the brain and have long-lasting impacts on behavior. Specifically, adolescent stress impairs hippocampal neurogenesis and can increase risk for anxiety, depression, and a dysregulated stress response in adulthood. In order to model the functional effects of reduced hippocampal neurogenesis during adolescence, a transgenic neurogenesis ablation rat model was used to suppress neurogenesis during the adolescent period and test anxiodepressive behaviors and stress physiology during adulthood. Wildtype and transgenic (TK) rats were given valganciclovir during the first two weeks of adolescence (4-6 weeks old) to knock down neurogenesis in TK rats. Starting in young adulthood (13 weeks old), blood was sampled for corticosterone at several time points following acute restraint stress to measure negative feedback of the stress response, and rats were tested on a battery of anxiodepressive tests at baseline and following acute restraint stress. Although TK rats had large reductions in both cell proliferation during adolescence, as measured by bromodeoxyuridine (BrdU), and ongoing neurogenesis in adulthood (by doublecortin), resulting in decreased volume of the dentate gyrus, negative feedback of the stress response following acute restraint was similar across all rats. Despite similar stress responses, TK rats showed higher anxiety-like behavior at baseline. In addition, only TK rats had increased depressive-like behavior when tested after acute stress. Together, these results suggest that long-term neurogenesis ablation starting in adolescence produces hippocampal atrophy and increases behavioral caution and despair amid stressful environments.

## Introduction

Stressful and traumatic experiences in early life increase the risk for developing anxiety and depressive disorders in adulthood (Penza et al., 2003; Lähdepuro et al., 2019), pointing to juvenile and adolescent development as sensitive periods for the influence of stress. Because human studies primarily rely on retrospective and correlational methods to link early life stress to adult dysfunction, animal studies are often used to model psychiatric conditions with more control over early life experiences (Belzung and Lemoine, 2011). The most reliable long-term effects into adulthood occur with neonatal or early postnatal stress, often involving maternal separation (Kalinichev et al., 2002). More variable are the effects of stress after weaning during the late juvenile or adolescent period. A variety of physical or social stressors during the early adolescent period can increase the occurrence of anhedonia, learned helplessness, defensive behaviors, and threat and novelty avoidance, all indicators for depression and anxiety in adult rodents (Wilkin et al., 2012; Chaby et al., 2015; Li et al., 2015; Cotella et al., 2019; Xu et al., 2019). However, many studies fail to find long-lasting effects of adolescent stress on adult hypothalamus-pituitary-adrenal (HPA) axis functioning (reviewed in McCormick et al., 2010). The studies that do have long-lasting effects of adolescent stress show adult HPA axis stress profiles related to that of depression, including enhanced baseline levels of corticosterone (Uys et al., 2006), and impaired negative feedback following either a dexamethasone test (Li et al., 2015) or acute stress (Isgor et al., 2004). The variability in adolescent stress effects on adult physiology and behavior could be due to the variability in the severity and type of stressors and age ranges used in different studies (McCormick et al., 2010), making purely behavioral studies difficult to directly compare.

One brain region particularly important for affective behaviors and normal stress reactivity is the hippocampus (Cameron and Glover, 2015). As the hippocampus goes through pronounced structural changes during the adolescent period (Bayer, 1982), the effects of adolescent stress on hippocampal structure may have pronounced effects on its adult functioning. Related to negative feedback of the stress response, adolescent stress downregulates glucocorticoid receptors on hippocampal neurons, a decrease still noticeable in adulthood (Isgor et al., 2004; Uys et al., 2006; Xu et al., 2019), making the hippocampus less sensitive to rising corticosterone levels and less able to provide negative feedback on the HPA axis. In addition to desensitization to corticosterone, hippocampal volume loss persists into adulthood across all major subregions (Isgor et al., 2004). Volume loss can reflect many structural mechanisms, but long-term changes to neuronal structure following adolescent stress have not been studied until very recently, with the demonstration of dendritic atrophy of CA3 neurons (Ghalandari-Shamami et al., 2021). Studies focusing on neurogenesis in the dentate gyrus region of the hippocampus largely find that adolescent stress increases cell proliferation and neurogenesis once in adulthood (Toth et al., 2008; McCormick et al., 2012). However, the few studies that measure the effects of stress on adolescent neurogenesis are mixed (Mouri et al., 2018; Harris et al., 2022). As neurogenesis is substantially higher during adolescence than adulthood (He and Crews, 2007), alterations to adolescent neurogenesis by stress have the potential to strongly alter the late development of the hippocampus and its structure and function into adulthood.

Multiple methods for neurogenesis suppression are available. During adulthood, irradiation, which eliminates neural precursor cells, prevents the decrease in depressive behaviors from antidepressant treatment (Santarelli et al., 2003; Surget et al., 2008; David et al., 2009) but does not typically increase anxiety-like or depressive behaviors on its own (Santarelli et al., 2003; Surget et al., 2008; David et al., 2009; Revest et al., 2009; Snyder et al., 2011). More recently, pharmacogenetic models have been utilized to directly target the generation of new neurons. One, the GFAP-TK model, uses a glial fibrillary acidic protein (GFAP) promoter to transgenically express a viral gene, herpes simplex virus thymidine kinase (TK), in precursor cells, which are then eliminated by an antiviral drug when they actively attempt to divide (Snyder et al., 2016). Transgenic (TK) mice with ablated adult hippocampal neurogenesis show impaired negative feedback to acute stress and display more anxiety-like and depressive behaviors in stressful conditions (Snyder et al., 2011), suggesting a role for new neurons of the hippocampus in stress reactivity. Relatedly, recent experiments demonstrate that reduction of new hippocampal neurons in adulthood prevents rodents from exhibiting behavioral flexibility from stress either naturally or following antidepressant treatment (Alves et al., 2017; Schoenfeld et al., 2019; Waters et al., 2022), despite different methods of neurogenesis suppression. Together, research suggests that adult neurogenesis in the hippocampus is most important for regulating emotional behavior amid changing environments (Cameron and Schoenfeld, 2018).

Ablating hippocampal neurogenesis during the adolescent period is less common. One study found that irradiation of adolescent mice produced no change in baseline behavior but made mice more reactive to foot shock stress in adulthood (Hayashi et al., 2008). Reduction of cell proliferation during the first two weeks of adolescence using the chemotherapy drug, methylazoxymethanol acetate (MAM), increased anxiety-like behavior in young adulthood – an effect that was rescued by enrichment during the same time period (Guo et al., 2013). Lastly, using a GFAP-TK model, mice with neurogenesis inhibition limited to early adolescence did not have lasting changes to depressive behaviors in adulthood but were more susceptible to social defeat (Kirshenbaum et al., 2014). Alterations to adolescent neurogenesis have the profound potential to dynamically alter the hippocampus and how neurons respond to stress (Kozareva et al., 2019), therefore we sought to determine the long-lasting effects of adolescent inhibition of neurogenesis on hippocampal structure and emotional behavior in adulthood. Using a GFAP-TK model, we inhibited neurogenesis starting in early adolescence and tested adult rats on a variety of anxiety-like and depression-like tasks at baseline and following acute stress.

## Materials and Methods

### Experimental Animals and Design

**FIGURE 1:**
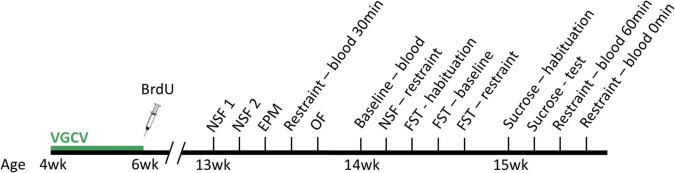
Experimental design. Wildtype and transgenic rats were given valganciclovir (VGCV) and concurrent bromodeoxyuridine (BrdU) injections during the first two weeks of adolescence, and were afterward undisturbed. Starting at 13 weeks old, all rats were tested on anxiodepressive tests at baseline and following acute restraint, including novelty-suppressed feeding (NSF), elevated plus-maze (EPM), open field (OF), sucrose preference, and forced swim (FST). Blood was collected at various times following the cessation of acute restraint stress to measure corticosterone levels as well. Rats were euthanized at 16 weeks old for histology.

Male transgenic Long-Evans rats were bred to express herpes simplex virus thymidine kinase (HSV-TK) under a glial fibrillary acidic protein (GFAP) promoter. The antiviral drug, valganciclovir (VGCV; 2 mg/rat), when administered, interferes with DNA replication of cells expressing the HSV-TK transgene and prevents cell division in the GFAP-expressing radial glial cells that produce new neurons (Snyder et al., 2016). By contrast, VGCV given to wildtype rats produces no detectable effects. Transgenic (TK) rats and wild type (WT) littermates were bred in-house (NIMH) and administered VGCV in peanut butter balls twice weekly during the first two weeks of adolescence (4-6 weeks old) to reduce cell proliferation in TK rats, assessed by a single injection of bromodeoxyuridine (BrdU; 200 mg/kg; Figure 1) following the last VGCV dose. All rats were meal-fed regular chow (∼16 g/rat/day) from the time of weaning, given *ad libitum* access to water, and kept on a 12-h light/dark cycle (lights off at 9 am).

Young adult male rats for experimentation (*n* = 10 WT rats, *n* = 6 TK rats) were transferred to Belmont University at 11 weeks old. Rats were maintained on the same meal-feeding schedule with *ad libitum* access to water, and a 12-h light/dark cycle (lights on at 6 am). Rats were given one week to acclimate following shipping and were handled for another full week until behavioral testing began at 13 weeks old. Over the period of 3 weeks, rats were tested on a battery of depression-like and anxiety-like behaviors at baseline (elevated plus-maze, open field, novelty-suppressed feeding, sucrose preference, and forced swim) and two additional tests after 30 min of restraint stress (novelty-suppressed feeding and forced swim). In addition, blood was collected on separate days at baseline and following different time periods after acute restraint stress to construct a corticosterone time course following acute psychological stress. At 16 weeks old, all rats were sacrificed and stained with BrdU to measure cell survival from early adolescence, doublecortin (DCX) to measure ongoing neurogenesis during young adulthood, and Nissl stains to assess dentate gyrus volume.

### Stress and Corticosterone

Because inhibiting hippocampal neurogenesis affects negative feedback of the HPA axis response (Snyder et al., 2011), tail veins were pricked and blood was extracted on separate days at different time points following 30-min of restraint stress (baseline no stress and at 0, 30, and 60 min following the cessation of restraint). Blood was centrifuged, plasma was collected, and reacted for corticosterone using an ELISA kit (Enzo Life Sciences Cat# ADI-900-097, RRID:AB_2307314) and analyzed on a 1420 multiplate reader (PerkinElmer). Rats were restrained in Plexiglas restraint tubes (Harvard Apparatus) at the same time each day (starting at 9 am) in a brightly lit room under the context of a different odor for each session to prevent habituation toward restraint tubes (ginger, peach, apple, banana cream, clove, and cinnamon; LorAnn Oils, Cat# 0444, 0450, 0350, 0250, 0080, and 0010; Schoenfeld et al., 2017).

### Anxiety-Like Behaviors

Anxiety-like behavior was assessed on the elevated plus-maze (EPM), open field (OF) and novelty-suppressed feeding (NSF) tests. After every trial, each apparatus was cleaned with 70% ethyl alcohol. The EPM was a plus-shaped course (65 cm off the ground, 40 cm x 10 cm arms) with two open arms and two closed arms with 40 cm-high walls. Testing was performed in a brightly lit room (250 lux). Rats were placed in the central section of the maze and exploratory behavior was observed for 5 min for each rat. The number of entries into the closed and open arms was recorded live as well as time spent in the open arms. A shorter period of time spent in the open arms was indicative of increased anxiety while total arm entries measured general locomotion.

The OF (1 m × 1 m) had a gray Plexiglas and reflective floor, marked with a 5 × 5 grid of lab tape under bright levels of light (250 lux) and scented with rosemary (LorAnn Oils, Cat# 2420). Rats were individually placed in a random corner of the OF and freely explored for 10 minutes. Entries into and through the 3 × 3 central grid of the OF as well as total grid entries throughout the OF were measured live. Less movement through the center was indicative of increased anxiety while total entries measured general locomotion.

The same arena as OF was used for NSF, with separate contextual elements to maintain novelty. For baseline NSF trials, the floor was covered with a thin layer of fresh wood chip bedding, walls were covered in laminated paper (color and patterns), lavender scent was used to contextualize the arena (LorAnn Oils, Cat# 2,270), the arena was placed under dim light levels (5 lux), and a small circle of white paper was placed in the center of the arena with one standard food pellet on top. Rats were food deprived for 24 h before testing and were all individually placed in the same starting corner. The time to first approach and then begin eating the food pellet in the center was scored live, with a maximum trial time of 10 min allowed. Three trials on NSF were run per rat total but on separate days. The first two trials were run with the above contextual elements in the arena to assess habituation of anxiety-like behavior with multiple trials. Because acute stress differentially changes behavior in the NSF in TK mice (Snyder et al., 2011), the third trial was run 30 min following cessation of 30 min of acute restraint (see above) and the arena was changed with new contextual elements, including smooth sandpaper floor, different wall decorations, and lemon scent (LorAnn Oils, Cat# 0020), but with the same light levels.

### Depression-Like Behaviors

Depression-like behavior was assessed on the sucrose preference test (SPT) and forced swim test (FST). SPT was conducted as previously described (Snyder et al., 2016). In short, rats were habituated to the presence of two water bottles, one containing 1% sucrose solution (Millipore Sigma, Cat# S8501) and one containing regular water over 4 days, alternating bottle position each day. On the day of testing, rats were water deprived for 8 h, then individually tested in a new cage with previously used bedding for a 10 min SPT. Sucrose preference was assessed by determining the percent drank by weighing water bottles before and after the test.

Forced swim was assessed as previously described (Slattery and Cryan, 2012). Rats were initially placed in a clear Plexiglas cylindrical tank (44 cm high, 18 cm diameter; Lafayette Instruments) filled with room temperature water to a height of 30 cm for 15 min to train learned helplessness as the tank is inescapable. On successive days, rats were placed into the water tank for 5 total minutes and at every 10 s interval, behavior was assessed as either immobile (floating), climbing (swimming up the walls), or swimming (swimming laterally or diving) with increased counts of immobility indicative of increased depression. The first test trial was considered a baseline trial and on the second test trial, rats were placed in the water tank 30 min following cessation of 30 minutes of restraint stress (see above).

### Immunohistochemistry

Brains were drop-fixed in 4% paraformaldehyde (Millipore Sigma, Cat# P6148) in 0.1M PBS following rapid decapitation at 4°C for 24 h. Brains were transferred into 20% sucrose solution at 4°C for 48 h before being frozen with dry ice and one hemisphere being sliced with a sliding microtome (American Optical). Brain sections were sliced coronally throughout the hippocampus at 40 μm on a 1:12 series before being stored in 0.1M PBS at 4°C. Each series contained 9-10 slices of hippocampal tissue from the anterior to posterior end and one full series was stained and analyzed for each histological measure.

To measure adolescent cell proliferation in the dentate gyrus of the hippocampus, a slide-mounted immunohistochemistry reaction for BrdU was conducted. Tissue was incubated in citric acid for 30 minutes at 90°C, rinsed in PBS, then mounted onto Superfrost Plus slides (ThermoFisher Scientific). Sections were then digested in trypsin (1 mg/mL 0.1M TB and 10% CaCl^2^, Millipore Sigma, Cat# T1426) for 10 min, rinsed, denatured in 2N HCl:PBS for 30 min, rinsed and incubated in primary antibody with 10% Tween-20 (1:200 mouse anti-BrdU; BD Biosciences Cat# 555627, RRID:AB_10015222) overnight at 4°C. The next day, tissue was rinsed, processed using a biotinylated ABC kit (Vector Laboratories Cat# PK-4001, RRID:AB_2336810; secondary for 60 min, AB for 60 min), then reacted with metal-enhanced DAB (ThermoFisher Scientific, Cat# 34065) for 10 min. Slides were counterstained with cresyl violet (as described below) and all BrdU+ cells were counted using a CX41 light microscope (Olympus) at 100x. Raw counts were multiplied by 24 to estimate for the whole hippocampus, bilaterally.

To measure ongoing hippocampal neurogenesis during young adulthood, a free-floating immunofluorescent reaction for DCX was conducted. Tissue was blocked in PBS-plus (0.1M PBS with 0.5% Tween-20 and 3% normal donkey serum) for 20 min before incubated in primary antibody in PBS-plus (1:500 rabbit anti-DCX; Cell Signaling Technology Cat# 4604, RRID:AB_561007) while shaken at 4°C, for 48 h. Tissue was then rinsed, incubated with fluorescent secondary antibody (1:500 AlexaFluor 555 donkey anti-rabbit; ThermoFisher Scientific, Cat# A-31572), counterstained with Hoescht 33,258 (1:1000; ThermoFisher Scientific, Cat# H3569), mounted, and coverslipped with ProLong Glass (ThermoFisher Scientific, Cat# P36980). All DCX+ cells were counted using an epifluorescent BX51 microscope (Olympus) at 40x. Raw counts were multiplied by 24 to estimate for the whole hippocampus, bilaterally.

To assess the volume of the dentate gyrus, a Nissl stain was conducted. Sections were mounted on Superfrost Plus slides (ThermoFisher Scientific, Cat# 22-037-246), rinsed, incubated in 0.5% cresyl violet (Millipore Sigma, Cat# C5042) for 6 min, dehydrated in 70% ethyl alcohol with acetic acid for 3 min, then 95 and 100% ethyl alcohol for 5 min. Lastly, slides were cleared in CitraSolv (Decon Laboratories, Cat# 1601) for 5 min and coverslipped using Permount (ThermoFisher Scientific, Cat# SP15). Using a CX41 light microscope, images were taken at 4x covering each section of dentate gyrus and section areas were assessed by tracing in ImageJ. Total estimated volume of the dentate gyrus was then estimated by adding the areas of 2D dentate gyrus tracings from one series of sections and converting to bilateral volume of the dentate gyrus.

### Statistical Analyses

All statistical analysis was conducted using Prism 8 (GraphPad Software). For EPM, OF, SPT, BrdU, DCX, and dentate gyrus volume, independent-samples *t*-tests were performed. For NSF, FST, and corticosterone, 2-way mixed factorial ANOVAs were performed with Bonferroni corrections for multiple comparisons used for interactions or main effects for variables with more than two conditions. To reduce possible Type I errors from multiple behavioral tests using the same animals, a Bonferroni correction for alpha level was applied to multiple data analyses from the same behavioral test. For all histology and sucrose preference data, α = 0.05, for open field, elevated plus-maze, and forced swim data, α = 0.025, for novelty-suppressed feeding data, α = 0.017, and for corticosterone measurements, α = 0.01.

## Results

### Knockdown of Adolescent Neurogenesis Negatively Affects Hippocampal Structure in Adulthood

**FIGURE 2:**
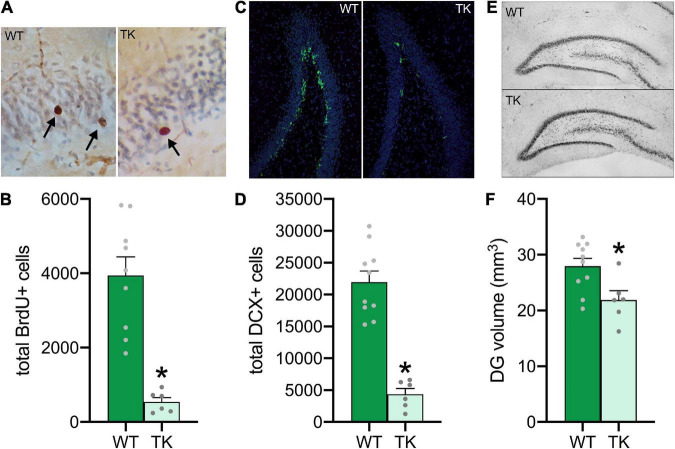
Knockdown of adolescent neurogenesis negatively affects hippocampal structure in adulthood. **(A)** Representative images of BrdU-labeled cells at 40x zoom. **(B)** Transgenic (TK) rats had cell proliferation inhibited during adolescence. **(C)** Representative images of DCX-labeled cells at 10x zoom. **(D)** TK rats had decreased neurogenesis during adulthood. **(E)** Representative images of Nissl stains at 4x zoom for dentate gyrus volume estimation. **(F)** TK rats had decreased volume of the dentate gyrus. *p < 0.05 compared to WT. All bars represent S.E.M. and dots represent individual rat data.

To verify that VGCV inhibited cell proliferation during the first two weeks of adolescence, adult brains were stained with BrdU (Figure 2A). TK rats had approximately 90% fewer BrdU-labeled cells in the adult dentate gyrus (*t*(14) = 5.43, *p* < 0.001), indicating a near-complete reduction in neurogenesis during adolescence (Figure 2B). To determine whether neurogenesis levels in TK rats rebounded to control levels in adulthood, adult brains were stained for immature neuron marker, DCX (Figure 2C). Despite cessation of VGCV treatment at 6 weeks old, TK rats still showed a roughly 80% reduction in DCX-labeled cells at 16 weeks old (*t*(14) = 7.41, *p* < 0.001), suggesting that adolescent knockdown of neurogenesis persisted into adulthood (Figure 2D). Because inhibition of neurogenesis in TK rats reduces the overall volume of the dentate gyrus in adulthood (Schoenfeld et al., 2017), dentate gyrus volume was assessed using tracing of Nissl-stained tissue at 16 weeks old (Figure 2E). With ongoing inhibition of neurogenesis from adolescence, TK rats had a 20% reduction in dentate gyrus volume (*t*(14) = 2.74, *p* = 0.02; Figure 2F).

### Inhibition of Neurogenesis From Adolescence Increases Stress-Associated Anxiodepressive Behaviors

**FIGURE 3:**
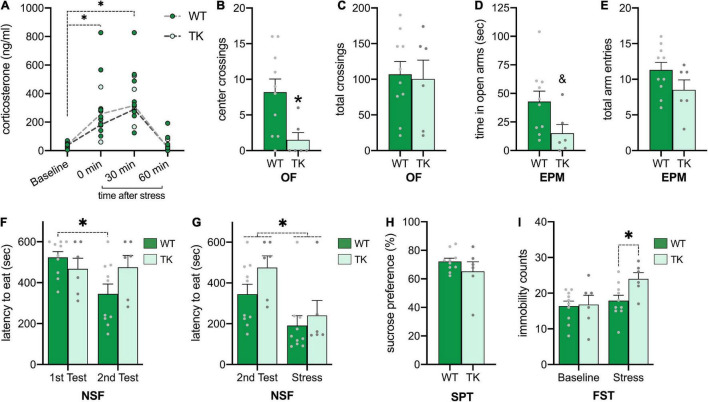
Inhibition of neurogenesis from adolescence increases stress-associated anxiodepressive behaviors. **(A)** Corticosterone is increased similarly following acute restraint **(B,C)** Transgenic (TK) rats had increased anxiety-like behavior on the open field **(B)**, despite similar locomotion **(C)**. **(D,E)** TK rats had a tendency toward decreased exploration in open arms **(D)** but similar overall locomotion in the elevated plus-maze **(E)**. **(F,G)** TK rats fail to habituate to the novelty-suppressed feeding arena at baseline **(F)**, but acute restraint hastens eating in all rats **(G)**. **(H)** All rats show similar sucrose preference. **(I)** All rats showed similar immobility in the forced swim test, but TK rats had more immobility following acute restraint. * *p* < α compared to baseline or wildtype (WT). & *p* < 0.10 compared to WT. All bars represent S.E.M. and dots represent individual rat data.

Because negative feedback of the HPA axis is impaired in adult TK mice with inhibited neurogenesis (Snyder et al., 2011), we measured corticosterone levels at various time points following acute restraint (Figure 3A). A main effect of time (*F*_3,39_ = 25.34, *p* < 0.001) showed that for all rats, corticosterone was elevated following restraint and returned to baseline 60 minutes after restraint. There was no main effect of genotype (*F*_1,13_ = 0.54, *p* = 0.48), nor time x genotype interaction (*F*_3,39_ = 0.50, *p* = 0.68), suggesting no differential effects on restraint-induced corticosterone in TK rats.

In addition to physiological effects of restraint, we tested the anxiety-like behavior of WT and TK rats in stress-inducing environments. In the OF (Figures 3B,C), TK rats had fewer crossings of the center grid (*t*(14) = 2.64, *p* = 0.02), despite similar exploration throughout the arena (*t*(14) = 0.21, *p* = 0.83), suggesting increased anxiety-like behavior. In the EPM (Figures 3D,E), results were similar. TK rats showed a tendency toward less time in the open arms (*t*(14) = 2.09, *p* = 0.05) but similar total arm entries (*t*(14) = 1.59, *p* = 0.14), suggesting more cautious and anxious behavior. Lastly, in the NSF all rats displayed high anxiety-like behavior by taking a long time to eat the food pellet, so rats were tested a second time to measure habituation to this test (Figure 3F). A genotype x test interaction (*F*_1_,_14_ = 9.78, *p* < 0.01) showed that WT rats habituated to the second test by decreasing time to eat the food pellet (*p* < 0.01), yet TK rats maintained similar anxiety-like behavior on the second test (*p* = 0.99). To then test the effects of stress on feeding behavior in the NSF, a third trial was conducted following acute restraint (Figure 3G). A main effect of restraint (*F*_1_,_14_ = 13.63, *p* < 0.01) showed that all rats ate more quickly following acute restraint, with no genotype (*F*_1,14_ = 2.15, *p* = 0.16) or interaction effects (*F*_1,14_ = 0.46, *p* = 0.58).

Lastly, because TK mice and rats have shown increased depressive behavior compared to WTs (Snyder et al., 2011, 2016), rats were tested for anhedonia in the SPT and for learned helplessness in the FST. In the SPT (Figure 3H), rats had similar sucrose preference in a 10-min test (*t*(13) = 1.18, *p* = 0.26). Rats were tested twice in the FST, once at baseline and then following acute restraint, after a learning trial (Figure 3I). A genotype x restraint interaction (*F*_1.14_ = 6.76, *p* = 0.02) suggested that at baseline, WT and TK rats had similar counts of immobility (*p* = 0.99), however following restraint, TK rats were more immobile than WT rats (*p* < 0.05).

## Discussion

Inhibition of neurogenesis starting in adolescence and persisting into adulthood caused significant atrophy of the adult dentate gyrus in neurogenesis-deficient rats. Despite these structural deficits, adult rats had relatively similar physiological stress responses to acute restraint. However, TK rat behavior suggested greater caution and despair, particularly in stressful environments. In anxiogenic environments, TK rats generally displayed more avoidance of stressful stimuli. In classic depressive-like behavior tests, TK rats behaved similarly to controls, but showed more learned immobility only following acute restraint. Together, the results suggest that inhibiting hippocampal neurogenesis starting in adolescence produces generalized cautious behavior across many environments, to a broader degree than in standard adult ablation models.

### Continuation of Neurogenesis Loss Into Adulthood

Although we intended to ablate neurogenesis only during the adolescent period, and gave VGCV only four times over two weeks at substantially lower doses than we have used in adult rats (Snyder et al., 2016), we were surprised to find that inhibition of neurogenesis in adolescence carried over into young adulthood. Our lab has previously shown partial recovery of neurogenesis, measured by DCX, following cessation of VGCV in adulthood (Snyder et al., 2016). Additionally, another lab showed full recovery of cell proliferation, measured by BrdU, shortly after cessation of GCV during adolescence (Kirshenbaum et al., 2014). This same lab has also shown that pre-adolescent treatment with VGCV leads to long-term reduction in neurogenesis into adulthood, measured by DCX (Youssef et al., 2019), consistent with our findings. These seeming contradictions could reflect differences in new cell fate, as residual progenitors continue to produce glia at high rates following inhibition of neurogenesis (Monje et al., 2002; Youssef et al., 2019). Although it seems that neurogenic recovery is possible in some studies, the conditions for this remain unclear.

Either way, suppression of neurogenesis from adolescence to young adulthood was enough to reduce the volume of the dentate gyrus by 20%, less than irradiation effects in juvenile mice (Naylor et al., 2005), but more than sustained VGCV treatment starting in adult rats (Schoenfeld et al., 2017). These structural changes are also likely linked to physiological changes as well. Loss of neurogenesis in the dentate gyrus not only alters network activity within the dentate gyrus (Lacefield et al., 2012), but feeds forward onto the rest of the hippocampal formation as well. Ablation of adult neurogenesis produces atrophy of pyramidal neurons in CA3 (Schoenfeld et al., 2017), and changes CA3 activity patterns to environmental stimuli and induced LTP within Schaeffer collaterals (Niibori et al., 2012; Glover et al., 2017; Sakalem et al., 2017). Although we only measured large-scale volume loss within the dentate gyrus, it is likely that more extensive effects throughout the hippocampus were produced through inhibition of adolescent neurogenesis.

### Knockdown of Neurogenesis Starting in Adolescence Produces More Extensive Behavioral Change

Arrest of hippocampal neurogenesis does not usually affect adult depressive behavior at baseline, either when ablation occurs in adulthood or transiently during adolescence (Santarelli et al., 2003; David et al., 2009; Snyder et al., 2011; Kirshenbaum et al., 2014), with some exceptions like decreased sucrose preference in adult TK rats (Snyder et al., 2016). However, following acute restraint, depressive behavior is increased to a larger degree in mice with deficient neurogenesis (Snyder et al., 2011). Likewise, our rats with inhibition of neurogenesis starting in adolescence were unaffected on sucrose preference and immobility in the forced swim at baseline but had enhanced immobility when tested following acute restraint. Given similar phenotypes to adult models, it is unclear if inhibiting neurogenesis in adolescence alters hippocampal functioning related to depression, compared to adult manipulations.

By contrast, our rats with inhibited neurogenesis starting in adolescence had stronger effects on adult anxiety-like behavior. TK rats had increased anxious behavior in adulthood, with decreased exploratory activity in anxiogenic parts of the open field and elevated plus-maze and deficient habituation of hyponeophagia in repeated tests of novelty-suppressed feeding. Increased anxiety-like behavior has been shown following ablation of hippocampal neurogenesis (Revest et al., 2009), but is normally unaffected in ablated adults (David et al., 2009; Snyder et al., 2011, 2016; Schoenfeld et al., 2016, 2019; Glover et al., 2017). During adolescence, mice given MAM to reduce neurogenesis show elevated anxiety-like behavior in adulthood, but only when MAM treatment is in early adolescence, similar to our timeline of VGCV treatment (Guo et al., 2013). This suggests that adult neurogenesis largely does not affect anxiety-like behavior, but early adolescent neurogenesis is necessary for normal anxiety-like behavior in adulthood. Considering that stress during early adolescence increases anxious behavior in adulthood (Wilkin et al., 2012; Chaby et al., 2015; Cotella et al., 2019), this suggests that the effects of adolescent stress on anxiety could be caused by suppressive effects on neurogenesis.

These effects on anxiety in TK rats reflect a generalized caution in multiple environments, both novel and familiar. WT rats, by comparison, show more flexible anxiety – displaying caution when exploring or foraging in novel environments, but able to habituate when the environment is learned to be non-threatening. Rats without neurogenesis appear like those under chronic stress (Glasper et al., 2012), displaying highly anxious behavior across multiple tests and failing to habituate in the NSF test. The one exception is when all rats showed an anxiolytic effect of acute restraint on the NSF test, similar to a previous TK rat study (Snyder et al., 2016). Because the NSF task is not just about exploring a novel environment but actively choosing to eat within a novel environment, these effects may be related to stress effects on feeding behaviors (Ely et al., 1997) and not simply anxiety broadly.

Extended ablation effects for adolescent TKs to increased anxiety as well as depression suggests more widespread alterations to the hippocampus than occurs with adulthood ablation. It is possible that adolescent ablation leads to a larger reduction in neural stem cells and neurogenesis than adulthood and this greater loss could bias hippocampal activity toward novelty-avoidance. However, the overall length of time for neurogenesis inhibition before behavioral analysis for our study is similar to others using adults (Snyder et al., 2016; Schoenfeld et al., 2019). In addition, as the general level of quiescence for stem cells is similarly high during adolescence as adulthood (Hochgerner et al., 2018), it is likely that similar loss of stem cells occur with both adolescent and adult ablation.

Although we don’t know how many new neurons are lost in adolescent versus adult ablation, it may be more important *when* neurogenesis is altered in terms of developing circuits to extrahippocampal areas. The medial prefrontal cortex (mPFC) receives direct projections from the ventral hippocampus, a pathway that is important for regulating anxiety-like behavior (Adhikari et al., 2010; Schoenfeld et al., 2014). During early adolescence, there is a dynamic increase in developing projections from ventral hippocampus to mPFC, projections that become pruned during late adolescence and are important for generating inhibitory synaptic plasticity within the mPFC (Caballero et al., 2014; Pattwell et al., 2016). This suggests that the adolescent period is important for the development of this pathway that helps provide stress resilience and fear extinction through inhibition (Zimmermann et al., 2019), and likely anxious behavior as well. Although it is unknown how adolescent neurogenesis is involved in development of these projections, disruption of normal adolescent hippocampal development has the potential to alter how this pathway is setup and may lead to long-lasting changes in affective processing.

### Beyond Hypothalamus-Pituitary-Adrenal Axis Reactivity

These behavioral changes occur despite similar plasma corticosterone profiles following acute restraint. Although enhanced HPA reactivity was originally discovered in TK mice (Snyder et al., 2011), two recently published reports show a more complicated picture in rats. One finds increased HPA reactivity to stress in male TK rats (Silveira-Rosa et al., 2022), whereas the other finds similar HPA reactivity between male WT and TK rats (O’Leary et al., 2022), which our study also supports. The reason for these discrepancies is unknown but may be due to either strain differences, duration and type of stressor, or additional methodological concerns. One cause for variability in our study was that separate time points for corticosterone before and after restraint were tested in the same rats on different days, using new odor context to reduce habituation to restraint tubes. Although this may have affected HPA axis activation and negative feedback on repeated testing days, the very first trial when rats were naïve was used to measure corticosterone 30 minutes after cessation of restraint, when TK mice have previously been shown to have maximal differentiation from WTs (Snyder et al., 2011). A lack of genotype effects at this time point and all others in our TK rats suggest similar circulating corticosterone levels following restraint, despite increased immobility following restraint in neurogenesis-depleted rats.

Although acute restraint was not used in all behavioral tests, our results suggest that corticosterone levels were likely similar across all behavioral tests in WT and TK rats. One possibility for behavioral effects despite similar corticosterone profiles could be alterations to glucocorticoid receptors (GRs) in the hippocampus. Genetic knockdown of GRs leads to higher depressive behavior following stress (Ridder et al., 2005), suggesting that changes to GR levels may alter how neurons respond and bias behavior during ongoing stress. Likewise, phosphorylation of GRs is associated with increased anxiodepressive behavior (Gao et al., 2018), showing that just altering the sensitivity of GRs in the hippocampus can influence stress-related behavior. Although adult mice with ablated neurogenesis show no change in glucocorticoid receptor protein or mRNA expression (Tsai et al., 2015; Silveira-Rosa et al., 2022), adolescent effects are unknown.

Another possibility is that loss of new neurons in the dentate gyrus affects GABAergic signaling within the hippocampus, important for regulating affective behavior (Crestani et al., 1999; Zou et al., 2016). Ablation of neurogenesis decreases GABAergic synapses in the dentate gyrus (Singer et al., 2011), similar to the effects of stress (Schoenfeld and Gould, 2013). In addition, neurogenesis ablation increases dentate gyrus excitability (Ikrar et al., 2013), excitability that is associated with increased stress-induced anxiodepressive behavior (Liu et al., 2021). As disruption in the excitatory/inhibitory balance within the hippocampus relates to anxiety-like and depressive behavior (Barbosa Neto et al., 2012; Alaiyed et al., 2020), any change to the relative contribution of glutamatergic and GABAergic transmission through loss of new neurons has the potential to bias hippocampal activity toward caution and despair.

### Limitations

In addition to hippocampal neurogenesis being ablated, the GFAP-TK rat model inhibits the generation of new neurons that populate the olfactory bulb (Snyder et al., 2016), although we did not directly test this in our rats. This is a possible limitation as we are not currently able to control for rates of olfactory neurogenesis using this pharmacogenetic model. None of the behavioral tasks WT and TK rats performed required olfactory processing, however, we did use odor cues to separate contexts and prevent habituation to repeated arenas. Research shows that olfactory neurogenesis is not necessary for simple olfactory discrimination (Lazarini and Lledo, 2011) and we have previously shown that TK rats can easily detect and change behavior due to the presence of overt odors like ones that we used (Schoenfeld et al., 2021), so olfactory abilities should not have affected TK behavior. However, low olfactory bulb volume is associated with depression (Negoias et al., 2010) and olfactory bulbectomy is a rodent model of depression (Song and Leonard, 2005), suggesting that developmental alterations to the olfactory bulb, not just the hippocampus, may contribute to changes in affect and mood. New neurons in the olfactory bulb consist of multiple types of inhibitory interneurons (Lledo et al., 2006), whereas recent research suggests that specific ablation of glutamatergic, but not GABAergic neurons, in the olfactory bulb induce depressive behavior (Yuan et al., 2020), suggesting that the reduction of new olfactory neurons may be less important for healthy mood states than losing preexisting glutamatergic ones. However, it is unknown how developmental reductions in olfactory neurogenesis may affect preexisting circuitry within the olfactory bulb. Future studies utilizing methods to specifically target neurogenesis in only one brain region will be needed to fully dissociate neurogenic effects on anxiety and depression.

Another limitation of the present study is the use of only male rats. We chose male rats to maximize sample size in our litter group due to unbalanced male and female generation and wanting to avoid using multiple litter groups at different times (Lazic and Essioux, 2013). This exclusion of females though is particularly important as sex differences have been reported on the effects of developmental stress on adult anxiodepressive behavior (Goodwill et al., 2019; Lovelock and Deak, 2019), stress effects on new neuron generation, survival, and activation (Yagi and Galea, 2019; O’Leary et al., 2022), and behavioral consequences following neurogenesis ablation (O’Leary et al., 2022; Silveira-Rosa et al., 2022; Waters et al., 2022). Other studies have shown comparable effects of neurogenesis ablation across sexes (Huckleberry et al., 2018; Seib et al., 2018; Cope et al., 2020), and it is unknown how our adolescent manipulations would have affected adult anxiodepressive behaviors in female rats. Additionally, research suggests that phase of the estrous cycle matters for both environmental effects on neurogenesis and ablation effects on behavior (Pawluski et al., 2009; Yohn et al., 2020; Silveira-Rosa et al., 2022). As gender differences exist in anxiety and mood disorders, typically starting during adolescence (Hankin and Abramson, 1999), it is important for future studies to explore the effects of altering neurogenesis and hippocampal development during adolescence in females as well.

Similar breeding realities outside of our control meant that our sample was largely made up of WT rats with relatively few TKs. To practice appropriate reduction of experimental animals and to minimize confounds associated with multiple litters, we have avoided adding additional litters to this analysis. However, the low sample size, particularly for TKs, means that some of our behavioral analyses may have missed potential effects of adolescent ablation of neurogenesis and we are at risk for Type I errors at the same time (Sullivan et al., 2016). Because of this, we choose to be cautious in the interpretations and generalizability of our data and acknowledge that future research will need to be done with larger samples to reproduce and replicate our results.

### Conclusion

Overall, we report that a large decrease in hippocampal neurogenesis starting in adolescence have long-term effects on hippocampal structure and stress-sensitive behavior resembling anxiety and depression. Because stress can influence rates of adolescent neurogenesis (Mouri et al., 2018), it is important to characterize the effects of aversive experiences on hippocampal development during the adolescent period for its effect not just during adolescence but into adulthood as well. However, other stress paradigms during adolescence have produced null results on neurogenesis (Harris et al., 2022), and in general there is a lack of knowledge for how different types of stressors of various frequencies and severities impact adolescent neurogenesis. As adolescent stress can impact other forms of structural plasticity (Eiland and Romeo, 2013), the more we understand about how stress impacts the developing hippocampus and rest of the brain, the better we can understand the relative resilience and susceptibility that various stressors have during the adolescent period.

## Data Availability Statement

The raw data supporting the conclusions of this article will be made available by the authors, without undue reservation.

## Ethics Statement

The animal study was reviewed and approved by NIMH Animal Care and Use Committee.

## Author Contributions

RL, NJ, HC, and TS contributed to the conception and design of the study. RL, NJ, NF, and TS performed the data collection and statistical analysis. RL and NJ wrote the first draft of the manuscript. TS wrote the sections of the manuscript. All authors contributed to manuscript revision, read, and approved the submitted version.

## Conflict of Interest

The authors declare that the research was conducted in the absence of any commercial or financial relationships that could be construed as a potential conflict of interest.

## Publisher’s Note

All claims expressed in this article are solely those of the authors and do not necessarily represent those of their affiliated organizations, or those of the publisher, the editors and the reviewers. Any product that may be evaluated in this article, or claim that may be made by its manufacturer, is not guaranteed or endorsed by the publisher.
